# Delayed Splenial Artery Pseudoaneurysm After Angiographically Negative Intracerebral Hemorrhage: A Case Report

**DOI:** 10.7759/cureus.107293

**Published:** 2026-04-18

**Authors:** Kotaro Ueda, Takafumi Mitsutake, Keisuke Kadooka, Michihiro Tanaka

**Affiliations:** 1 Department of Neuroendovascular Surgery, Kameda Medical Center, Kamogawa, JPN; 2 Department of Neuroendovascular Surgery, Department of Neurosurgery, Kameda Medical Center, Kamogawa, JPN

**Keywords:** angiographically negative, delayed formation, endovascular treatment, nbca, neurointervention, parent artery occlusion, platelet disorder, posterior pericallosal artery, pseudoaneurysm, splenial artery

## Abstract

Intracranial pseudoaneurysms are rare vascular lesions and are associated with a high risk of rupture. Most reported cases are related to trauma or iatrogenic injury, whereas their association with non-traumatic intracerebral hemorrhage remains unclear. Here, we present a case of a 77-year-old female with essential thrombocythemia who presented with acute memory disturbance and headache. Computed tomography revealed an intracerebral hemorrhage in the left cingulate isthmus extending to the parietal lobe. Initial magnetic resonance imaging and digital subtraction angiography failed to identify the source of bleeding. Approximately one month later, during endovascular treatment for an incidental unruptured basilar tip aneurysm, repeat angiography revealed a pseudoaneurysm corresponding to the prior hemorrhagic site. Selective angiography demonstrated that the pseudoaneurysm originated from the splenial artery (posterior pericallosal artery). The pseudoaneurysm was successfully treated with n-butyl cyanoacrylate embolization with parent artery occlusion, followed by stent-assisted coil embolization of the basilar tip aneurysm. The patient was discharged without neurological deficits, and no recurrence was observed on six-month follow-up magnetic resonance imaging. This case highlights the potential for delayed formation of a pseudoaneurysm after non-traumatic intracerebral hemorrhage, particularly in atypical locations and possibly in patients with underlying platelet disorders. Repeat angiographic evaluation should be considered when the initial study fails to identify the bleeding source.

## Introduction

Intracranial pseudoaneurysms are rare entities, accounting for approximately one percent of all intracranial aneurysms, and are associated with high morbidity and mortality due to their propensity for rupture and rebleeding [1]. They are commonly associated with trauma, infection, or iatrogenic vascular injury following neurosurgical or neurointerventional procedures [1, 2], whereas spontaneous cases without an identifiable precipitating factor are relatively uncommon.

Pseudoaneurysms are characterized by complete disruption of the arterial wall, resulting in a hematoma cavity that communicates with the parent artery [1, 3]. Due to the absence of a normal vessel wall structure, they are fragile and have a high risk of rupture.

Although intracranial pseudoaneurysms can present with hemorrhage, their role in non-traumatic intracerebral hemorrhage remains unclear. In particular, delayed formation of pseudoaneurysms after an initial hemorrhagic event poses a diagnostic challenge, especially when initial angiographic studies are negative [4, 5].

We report a rare case of delayed pseudoaneurysm formation in the splenial artery (posterior pericallosal artery) following intracerebral hemorrhage in the cingulate isthmus and discuss the diagnostic challenges, pathophysiology, and treatment strategy.

## Case presentation

A 77-year-old right-handed female with a history of essential thrombocythemia, hypertension, and hyperuricemia presented with sudden-onset memory disturbance and headache. Her medications included anagrelide, nifedipine, and febuxostat. On admission, her blood pressure was 224/106 mmHg. Neurological examination revealed memory impairment and ideomotor apraxia without motor weakness, sensory disturbance, or aphasia. Laboratory findings showed no abnormalities in coagulation parameters, including a platelet count of 327 ×10⁹/L, indicating no apparent bleeding diathesis (Table 1). 

**Table 1:** Laboratory findings on admission Coagulation parameters and platelet count were within normal limits. PT - prothrombin time; INR - international normalized ratio

Parameter	Value	Reference range
Platelet count	327 ×10⁹/L	158–348 ×10⁹/L
PT (INR)	1.09	0.80-1.30
PT (%)	87.00%	70-120 %
Prothrombin time	12.1 sec	10.0-13.0 sec
Activated partial thromboplastin time	26.5 sec	20.0-38.0 sec
D-dimer	<0.5 μg/mL	0.0-1.0 μg/mL

**Figure 1:**
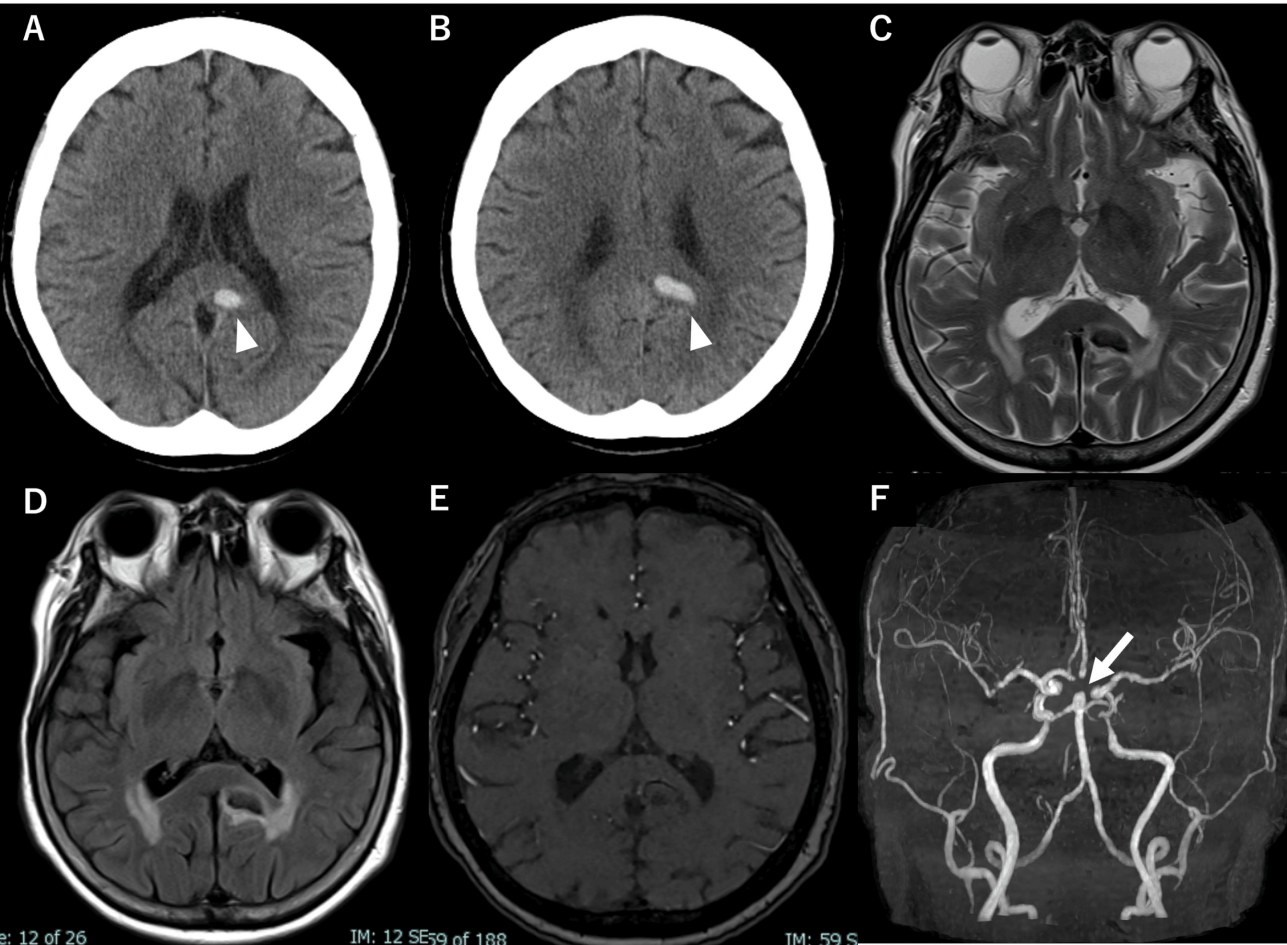
Initial imaging findings Non-contrast computed tomography (A, B) demonstrates intracerebral hemorrhage in the left cingulate isthmus extending to the parietal lobe (white arrowheads). Magnetic resonance imaging shows the hemorrhagic lesion on T2-weighted imaging (C) and fluid-attenuated inversion recovery imaging (D). No clustering of flow voids is observed around the hematoma on T2-weighted imaging, suggesting the absence of an underlying vascular malformation. Time-of-flight magnetic resonance angiography source image (E) demonstrates no abnormal vascular clustering surrounding the hemorrhagic lesion. Three-dimensional reconstructed magnetic resonance angiography (F) demonstrates an incidental basilar tip aneurysm (white arrow).

Computed tomography (CT) demonstrated intracerebral hemorrhage in the left cingulate isthmus extending to the parietal lobe (Figure 1A-B). Magnetic resonance imaging did not reveal vascular malformations or abnormal flow voids (Figure 1C-E). An incidental 7.2 mm basilar tip aneurysm was identified but considered unrelated to the hemorrhage (Figure 1F). 

**Figure 2:**
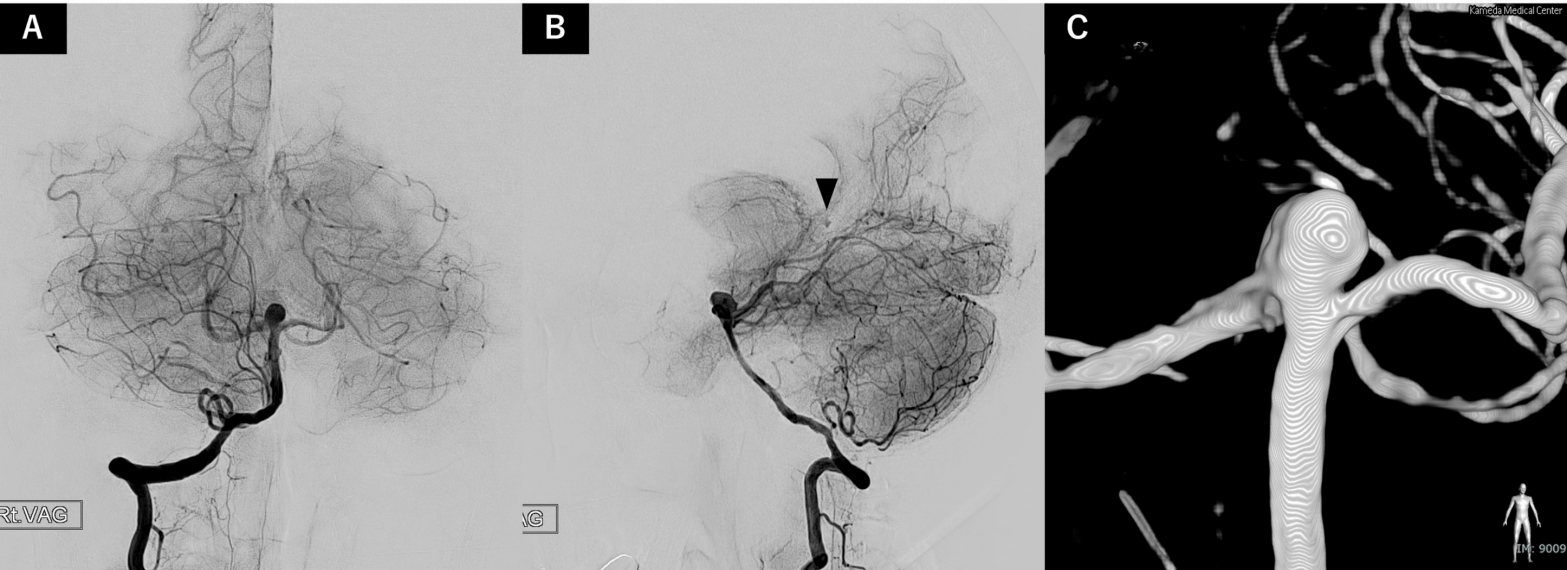
Initial angiographic evaluation Digital subtraction angiography performed seven days after onset demonstrates no definite source of bleeding. Delayed arterial phase images of right vertebral artery angiography are shown in the anteroposterior view (A) and lateral view (B). Retrospective review suggests a subtle abnormality suspicious for a pseudoaneurysm (black arrowhead), although no definitive angiographic findings were identified at that time. Three-dimensional rotational angiography (C) demonstrates an incidental 7.2 mm basilar tip aneurysm.

Digital subtraction angiography (DSA) performed seven days after onset failed to identify a definite bleeding source. However, retrospective review revealed a subtle abnormality suspicious for a pseudoaneurysm, although no contrast pooling or definitive angiographic features were observed at that time (Figure 2). 

The patient was managed conservatively and showed gradual neurological improvement. She was discharged 10 days after admission with a modified Rankin Scale score of zero. Approximately one month later, endovascular treatment for the basilar tip aneurysm was planned, and dual antiplatelet therapy was initiated.

The procedure was performed under general anesthesia. A right distal radial artery approach was initially attempted; however, severe radial artery spasm persisted despite repeated intra-arterial administration of vasodilators. Therefore, the approach was converted to a transfemoral route. A 6 Fr FUBUKI guiding sheath (Asahi Intecc, Aichi, Japan) was inserted via the right femoral artery and advanced into the left vertebral artery, and systemic heparinization was achieved.

**Figure 3:**
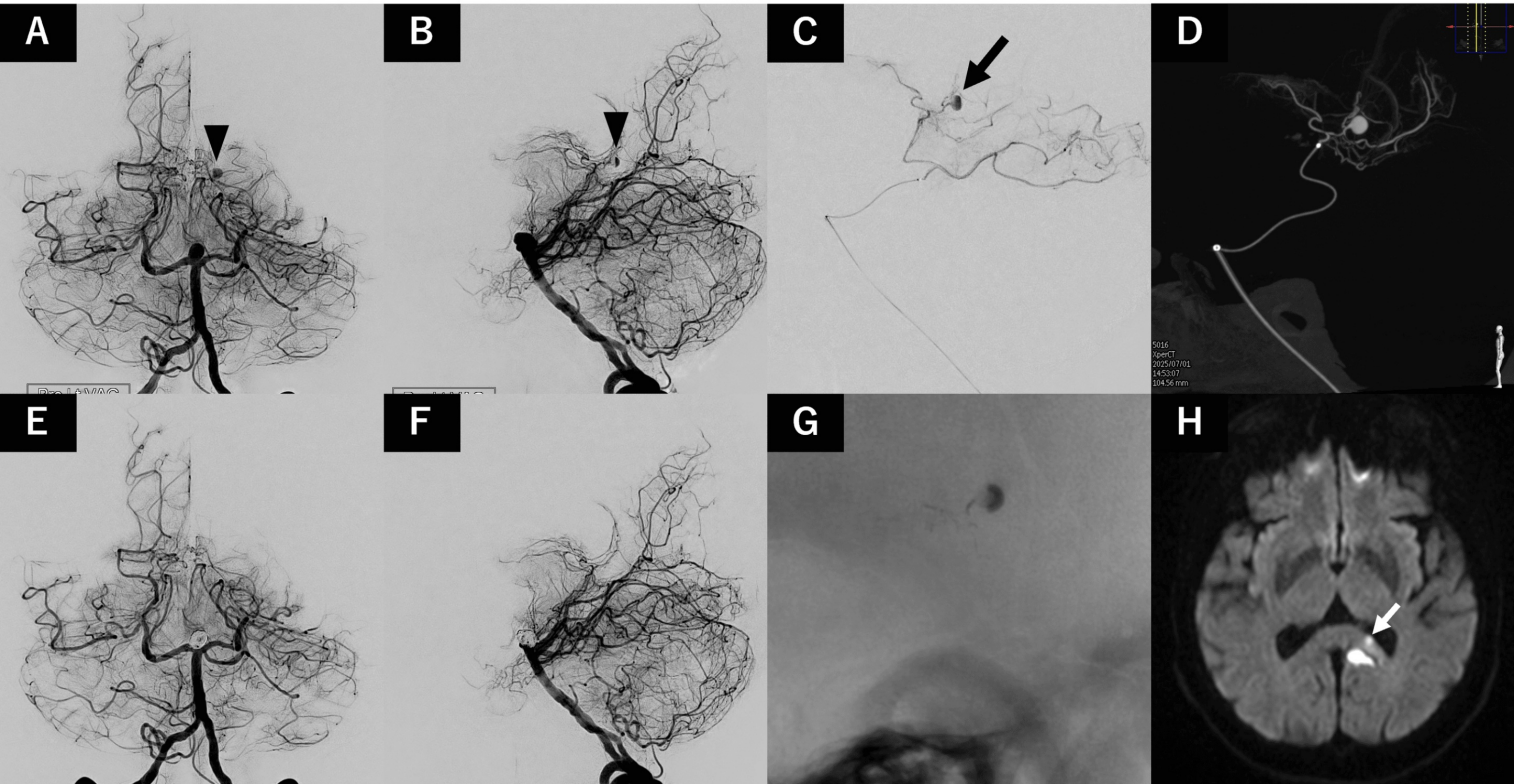
Endovascular treatment of the pseudoaneurysm Left vertebral artery angiography performed one month after the initial hemorrhage during treatment of the basilar tip aneurysm. Pre-treatment delayed arterial phase images are shown in the anteroposterior view (A) and lateral view (B), demonstrating a pseudoaneurysm arising from the splenial artery (black arrowheads). Selective angiography via a microcatheter (C) and cone-beam computed tomography (D) were performed to delineate the precise location of the pseudoaneurysm (arrow). Post-treatment angiography in the anteroposterior (E) and lateral (F) views demonstrates complete occlusion of the pseudoaneurysm with preservation of adjacent branches and near-complete occlusion of the basilar tip aneurysm. A lateral radiograph (G) demonstrates the glue cast, showing adequate penetration into the pseudoaneurysm. Postoperative diffusion-weighted magnetic resonance imaging (H) demonstrates a small spotty hyperintensity adjacent to the hemorrhagic lesion (white arrow).

Diagnostic angiography revealed a previously undetected 3.5 mm pseudoaneurysm arising from the splenial artery corresponding to the prior hemorrhagic site (Figure 3A-B). Based on this finding, priority was given to the treatment of the pseudoaneurysm due to the risk of rupture.

A distal access system consisting of a 3.2 Fr. intermediate catheter Guidepost (Tokai Medical Products, Aichi, Japan) and a 1.5 Fr. microcatheter Marathon (Medtronic, Minneapolis, MN, USA) was used. The intermediate catheter was advanced to the P1 segment of the left posterior cerebral artery, and the microcatheter was navigated distally.

Selective angiography and cone-beam CT confirmed the precise location of the pseudoaneurysm (Figure 3C-D). The microcatheter was positioned proximal to the origin of the splenial artery from the lateral posterior choroidal artery.

Parent artery occlusion was performed using 12.5% n-butyl cyanoacrylate under careful control to avoid reflux. The embolic material adequately penetrated the pseudoaneurysm, achieving complete occlusion (Figure 3E-G).

Following successful treatment of the pseudoaneurysm, stent-assisted coil embolization of the basilar tip aneurysm was performed using the jailing technique, achieving near-complete occlusion (Figure 3E-F).

Postoperative magnetic resonance imaging (MRI) demonstrated small, asymptomatic diffusion-weighted imaging hyperintensities in the splenium of the corpus callosum (Figure 3H). The patient was discharged without neurological deficits three days after the procedure. No recurrence was observed at the six-month follow-up MRI.

## Discussion

The present case demonstrates a rare instance of delayed pseudoaneurysm formation in the splenial artery following non-traumatic intracerebral hemorrhage in the cingulate isthmus. Notably, no apparent precipitating factors such as trauma, prior neurosurgical intervention, or infection were identified, suggesting a spontaneous mechanism. Spontaneous pseudoaneurysms without trauma or infection are uncommon but have been reported in association with underlying vascular fragility [6].

A key feature of intracranial pseudoaneurysms is their delayed formation. These lesions may not be detectable during the acute phase of hemorrhage and can become angiographically apparent days to weeks later [1, 4, 5]. In the present case, the initial DSA performed seven days after onset failed to demonstrate a definitive bleeding source. However, retrospective review revealed a subtle abnormality suspicious for a pseudoaneurysm, although no contrast pooling or characteristic angiographic features were observed to establish a definitive diagnosis at that time. The lesion became clearly visible on repeat angiography approximately one month later. This temporal evolution strongly supports the hypothesis that the pseudoaneurysm developed secondary to vessel wall disruption caused by the initial hemorrhage, followed by recanalization and progressive enlargement during the subacute phase, rather than representing the primary bleeding source.

The diagnosis of intracranial pseudoaneurysm is often challenging, particularly in the absence of histopathological confirmation. In clinical practice, the diagnosis is typically based on a combination of angiographic morphology and clinical course. Angiographically, pseudoaneurysms often demonstrate irregular or blister-like morphology, absence of a well-defined neck, delayed contrast filling and stagnation, and dynamic changes over time [1, 3, 5]. Another important feature is their delayed appearance after an initially negative angiographic study. In the present case, the initial angiography lacked definitive findings such as contrast pooling, making diagnosis difficult. However, the subsequent appearance of a clearly defined lesion corresponding to the hemorrhagic site strongly supports the diagnosis of a pseudoaneurysm.

Differentiating pseudoaneurysms from true aneurysms is also clinically important but often difficult without histopathological confirmation. True aneurysms typically have a well-defined neck and relatively stable morphology over time, whereas pseudoaneurysms tend to demonstrate rapid morphological changes and lack a structured arterial wall. Histopathologically, pseudoaneurysms are characterized by complete disruption of the arterial wall and formation of a cavity surrounded by hematoma or fibrous tissue [1]. However, such confirmation is rarely feasible in clinical practice unless surgical resection or autopsy is performed. In the present case, the delayed appearance after hemorrhage, irregular morphology, and lack of a clear neck were more consistent with a pseudoaneurysm than with a true aneurysm. Therefore, although histopathological confirmation was not obtained, the lesion was clinically and angiographically considered to represent a pseudoaneurysm.

Another noteworthy aspect of this case is the unusual location of the hemorrhage. To our knowledge, there are no previous studies specifically evaluating the causes of hemorrhage in the cingulate isthmus, and no data are available regarding the most common underlying etiologies in this region. Nevertheless, hemorrhage in the cingulate isthmus is not considered a typical location for hypertensive intracerebral hemorrhage. Therefore, when hemorrhage occurs in this region, secondary causes such as arteriovenous malformation, dural arteriovenous fistula, venous sinus thrombosis, or other vascular lesions should be considered. Although no vascular abnormality was identified on initial imaging, small vascular lesions such as micro arteriovenous malformations cannot be completely excluded, as they may be obscured by the hematoma or become angiographically occult because of disruption or thrombosis after rupture. In the present case, subsequent angiographic studies did not demonstrate any definite vascular abnormality other than the pseudoaneurysm. However, the possibility remains that a micro arteriovenous malformation or another tiny vascular lesion was initially present and that the pseudoaneurysm subsequently developed secondarily after the hemorrhagic event.

The anatomical origin of the pseudoaneurysm further supports its relationship to the hemorrhagic site. The splenial artery, a distal branch of the posterior pericallosal artery, supplies the splenium of the corpus callosum and adjacent cingulate isthmus. Distal artery pseudoaneurysms are rare but have been reported most commonly in the anterior cerebral artery and pericallosal territory [7, 8]. The lesion location in the present case is consistent with these previously reported distributions. However, to our knowledge, no previous reports have described a splenial artery pseudoaneurysm presenting after a cingulate isthmus hemorrhage, making this case exceptionally rare.

The possible contribution of essential thrombocythemia and its treatment should also be considered. The patient was receiving anagrelide for essential thrombocythemia. Although several reports have described intracranial hemorrhage in patients receiving anagrelide, these cases were frequently associated with concomitant antiplatelet therapy, making it difficult to establish a direct causal relationship with anagrelide alone [9]. In the present case, the coexistence of essential thrombocythemia and anagrelide may have promoted vascular fragility and impaired hemostasis. We speculate that these factors contributed not only to the initial hemorrhage but also to the subsequent formation of the pseudoaneurysm. Because hemorrhage in the cingulate isthmus is atypical and no definite vascular lesion was identified initially, impaired hemostasis associated with essential thrombocythemia and its treatment may have facilitated hemorrhage from a small distal vessel. Subsequent progressive disruption of the injured arterial wall may then have resulted in delayed pseudoaneurysm formation and enlargement.

From a therapeutic perspective, intracranial pseudoaneurysms are fragile lesions with a high risk of rupture, and direct clipping or selective coiling is often technically challenging. Although treatment strategies such as stent-assisted coiling and flow-diverter stent placement have been reported [1, 5, 7], parent artery occlusion is frequently considered an effective treatment option for distal lesions, particularly when sufficient collateral circulation is present. Liquid embolic agents such as N-butyl cyanoacrylate (NBCA) or Onyx have also been reported as useful treatment options in selected cases [1, 7, 10]. In the present case, parent artery occlusion was selected due to the distal location of the lesion. Although coil embolization was a possible alternative, the microcatheter could be advanced sufficiently close to the pseudoaneurysm, allowing effective penetration of NBCA. As anticipated, the glue cast penetrated not only the parent artery but also the pseudoaneurysm itself, resulting in complete occlusion. This approach highlights the utility of liquid embolic agents in the treatment of distal pseudoaneurysms and supports their use when adequate catheter positioning can be achieved. Delayed rebleeding due to pseudoaneurysm formation has also been reported, highlighting the importance of careful follow-up and timely intervention [11].

Angiographic evaluation is not necessary in all patients with intracerebral hemorrhage. Because hypertensive hemorrhage accounts for the majority of spontaneous intracerebral hemorrhage, routine angiography is generally unnecessary when the hemorrhage is located in typical sites of hypertensive hemorrhage, such as the putamen, thalamus, brainstem, or cerebellum. In contrast, angiographic evaluation should be considered when hemorrhage occurs in an atypical location, particularly in lobar, parasagittal, callosal, or cingulate regions, because these locations are more likely to be associated with underlying vascular lesions. CT angiography may serve as a practical and less invasive alternative to digital subtraction angiography, and pseudoaneurysms may sometimes be detected as focal contrast pooling within or adjacent to the hematoma. However, the spatial resolution of CT angiography is inferior to that of DSA for detecting very small vascular lesions. Therefore, a negative CT angiography study does not necessarily exclude the presence of a micro arteriovenous malformation, small dural arteriovenous fistula, or tiny distal pseudoaneurysm. In patients with persistent suspicion despite negative noninvasive imaging, repeat DSA after an interval should be considered.

This case highlights several important clinical implications. First, an intracranial pseudoaneurysm should be considered in patients with intracerebral hemorrhage of unknown origin, particularly when the hemorrhage occurs in an atypical location. Second, a negative initial angiographic study does not exclude the presence of a vascular lesion, and repeat angiographic evaluation is essential. Finally, careful interpretation of angiographic findings in conjunction with clinical course is critical for distinguishing pseudoaneurysms from other vascular pathologies and for determining the optimal treatment strategy.

## Conclusions

We report a rare case of delayed splenial artery pseudoaneurysm following cingulate isthmus hemorrhage. This case highlights the potential for delayed formation of a pseudoaneurysm after non-traumatic intracerebral hemorrhage, particularly in atypical locations and possibly in patients with underlying platelet disorders. Repeat angiographic evaluation should be considered when the initial study fails to identify the source of bleeding. Early identification and appropriate treatment may prevent rebleeding and improve clinical outcomes.
